# Notch1 promotes the pericyte-myofibroblast transition in idiopathic pulmonary fibrosis through the PDGFR/ROCK1 signal pathway

**DOI:** 10.1038/s12276-019-0228-0

**Published:** 2019-03-20

**Authors:** Yi-Chun Wang, Qiong Chen, Jun-Ming Luo, Jing Nie, Qing-He Meng, Wei Shuai, Han Xie, Jia-Mei Xia, Hui Wang

**Affiliations:** 1 Departerment of Critical Care Medicine, The Third Affiliated Hospital of Guangzhou Medical University, No. 63, Duobao Road, Guangzhou, 510150 P. R. China; 2 Department of Critical Care Medicine, Hunan Cancer Hospital, Changsha, 410013 P. R. China; 3 Department of Surgery, SUNY Upstate Medical University, Syracuse, NY 13210 USA; 4 Department of Thoracic Radiotherapy, Hunan Cancer Hospital, Changsha, 410013 P. R. China

**Keywords:** Cell biology, Molecular biology

## Abstract

The goals of this study were to investigate the role of the Notch1/PDGFRβ/ROCK1 signaling pathway in the pathogenesis of pulmonary fibrosis and to explore the possibility of treating fibrosis by targeting Notch1. Lung tissues from patients with pulmonary fibrosis were examined for the expression of Notch1/PDGFRβ/ROCK1 using RT-qPCR, western blotting, and immunostaining. Cultured mouse lung pericytes were transfected with Notch1-overexpressed vectors or shRNA targeting PDGFRβ/ROCK1 to examine cell behaviors, including proliferation, cell cycle arrest, and differentiation toward myofibroblasts. Finally, a mouse pulmonary fibrosis model was prepared, and a Notch1 inhibitor was administered to observe tissue morphology and pericyte cell behaviors. Human pulmonary fibrotic tissues presented with overexpression of Notch1, PDGFRβ, and ROCK1, in addition to a prominent transition of pericytes into myofibroblasts. In cultured mouse lung pericytes, overexpression of Notch1 led to the accelerated proliferation and differentiation of cells, and it also increased the expression of the PDGFRβ and ROCK1 proteins. The knockdown of PDGFRβ/ROCK1 in pericytes remarkably suppressed pericyte proliferation and differentiation. As further substantiation, the administration of a Notch1 inhibitor in a mouse model of lung fibrosis inhibited the PDGFRβ/ROCK1 pathway, suppressed pericyte proliferation and differentiation, and alleviated the severity of fibrosis. Our results showed that the Notch1 signaling pathway was aberrantly activated in pulmonary fibrosis, and this pathway may facilitate disease progression via mediating pericyte proliferation and differentiation. The inhibition of the Notch1 pathway may provide one promising treatment strategy for pulmonary fibrosis.

## Pulmonary fibrosis: Moving signals down a notch might help

A cell membrane protein called Notch1, which binds to signaling molecules outside cells and then alters the activity of genes inside the cells, might be a promising target for drugs to treat the lung damage of pulmonary fibrosis. This condition, generally of unknown cause, involves thickening, stiffening and scarring of lung tissue. It can lead to serious breathing difficulties and eventually death, especially in people aged over 70. Hui Wang and colleagues at Central South University, Changsha, investigated the significance of the Notch1 signaling pathway by examining lung tissue from patients and manipulating the activity of the pathway in mouse cells. They conclude that Notch1 signaling is activated in pulmonary fibrosis. Drugs that could inhibit the pathway, for example by binding to the Notch1 protein, might open a promising new avenue toward treatment.

## Introduction

Idiopathic pulmonary fibrosis (IPF) is characterized as interstitial pneumonia in adults with an unknown etiology^1^. The estimated prevalence of IPF is 50 cases per 100,000 people in the US, and the incidence rises sharply with age, especially in those older than 70 years^2^. IPF usually has an unfavorable prognosis and is unresponsive to classical medicines that treat fibrosis^3^. It is reported that the IPF patient population has only a 3-year median survival period, and the disease usually ends with respiratory failure^4^. More importantly, IPF patients may experience acute episodes of disease exacerbation for unknown reasons^5^, significantly elevating the death rate. It is thus important to further reveal the underlying cellular and molecular mechanisms of IPF for the diagnosis and development of medication.

The pericyte-myofibroblast transition (PMT) is characterized by the detachment of pericytes from endothelial walls, where they migrate into the interstitial space and undergo transition into myofibroblasts^6^. The newly colonized fibroblasts and consequently activated inflammatory macrophages further cause capillary fibrosis in multiple organs such as the kidney^6^. Pericytes have also been shown to have critical roles in myofibroblast activation in models of fibrotic inflammatory lung disease^7^. Recently, preventing the differentiation of pericytes into myofibroblasts has been proposed to be one potential strategy in modulating kidney fibrosis secondary to diabetic nephropathy^8^. Therefore, modulating PMT may be one promising treatment approach for IPF.

The Notch signaling pathway is widely known to be involved in pericyte differentiation. For example, Notch1 is known to induce pericyte differentiation into glioblastoma stem cells^9^, and Notch3 can maintain the pericyte populations in brain vessels^10^. A recent study found that DLL4, a potent Notch1 ligand, could stimulate the differentiation of primary cultured kidney pericytes into myofibroblasts^11^. Furthermore, the Notch pathway is also involved in the pathogenesis and progression of IPF. In rat pulmonary fibrosis, the levels of the Notch1 receptor and its ligand are elevated^12^. The targeting of the Notch signaling pathway can attenuate hepatic fibrosis in rats^13^. In summary, inhibition of the Notch pathway has been proposed as a potential treatment for tissue fibrosis^14^. A recent study showed that attenuation of the Notch1 signal in mesenchymal tissues alleviated pulmonary fibrosis^15^. Specifically, Notch1 is reported to stimulate vascularization by enhancing the expression of platelet-derived growth factor receptor β (PDGFRβ) in pericytes^9^. However, no evidence has been provided for the modulation of the PMT event by Notch via targeting the PDGFR pathway. Based on this information, we thus propose that the Notch1 signaling pathway may mediate the PMT event, which further plays a role in IPF.

PDGF can stimulate the growth, motility, and epithelial transition of mesenchymal cells^16^. Among the various isoforms of PGDFR, PDGFRβ has been implicated under various mesenchymal cell pathological conditions to induce disease, including pulmonary fibrosis^17^. Therefore, the investigation of signaling that is related to PDGFRβ is of critical importance for its intervention. Previous studies have shown that Notch1 may modulate PDGFRβ activity^9^. Upon its activation, PDGFRβ can modulate various downstream signaling molecules such as Ras, phosphatidylinositol 3-kinase (PI3K) and phospholipase C (PLC)^18, 19^. In addition, PDGFRβ also exerts modulatory roles on Rho-associated protein kinase 1 (ROCK1) to mediate the migration of vascular smooth muscle cells^20^. As ROCK1 is also involved in the pathogenesis of pulmonary fibrosis^21^, we thus speculate that PDGFRβ-ROCK1 has a role in IPF.

In this study, we performed both in vitro and in vivo studies to examine the effect of Notch1 and its related signaling pathway in the pathogenesis of pulmonary fibrosis. We showed that lung tissues from IPF patients had an elevated expression of Notch1 and its downstream effectors PDGFRβ and ROCK1. In cultured mouse lung pericytes, we found that Notch1-mediated cell proliferation and the differentiation toward myofibroblasts via PDGFRβ/ROCK1. With a mouse model, we further showed that a Notch1 inhibitor alleviated lung fibrosis, thus providing a potential treatment strategy for human IPF.

## Materials and methods

### Collection of lung tissue samples from IPF patients

A total of 26 IPF patients were diagnosed based on consensus guideline of pulmonary fibrosis without a known secondary etiology. Lung tissue samples were collected during surgery and were kept in liquid nitrogen. All human pulmonary tissues that were used in this study were provided by the Pathology Department of a Hunan Cancer Hospital. This study has been preapproved by the ethical committee of our hospital (license number: 201701), written consent was obtained from all participants.

### Primary pulmonary pericyte culture

The isolation and culture of mouse pericytes followed previously documented protocols^22^. Mouse lung tissues were harvested and incubated with proteinase and DNase in DMEM medium for 30 min at 37 °C. After digestion, the tissue lysate was filtered through 40 μm filters, centrifuged, and resuspended in cold PBS buffer with 2 nM EDTA. To separate the pericyte, cells were incubated with the anti-PDGFRβ polyclonal antibody (#3169, Cell Signaling Technology, US). After washing, cells were incubated in goat anti-IgG microbeads and were selected by MACS magnetic (Miltenyi Biotec, US) separation. The selected cells were kept in DMEM/F12 (Gibco, US) medium supplemented with 10% FBS (HyClone, US) plus 1% penicillin/streptomycin.

### Immunofluorescent staining

Purified pericytes were quantified using immunofluorescent staining. In brief, cells were cultured on cover slides and were fixed in 4% paraformaldehyde. Cells were treated with 0.1% Triton X-100 to increase the permeability of the membrane. After incubation in 3% normal goat serum (Invitrogen, US) for 1 h for blocking, cells were incubated overnight with anti-PDGFR-β (#3169, 1:100, Cell Signaling Technology, US), NG2 (SC-20162, 1:100, Santa Cruz, CA), CD13 (ab108382, 1:200, Abcam, US), or α-SMA (ab5694, 1:100, Abcam, US). On the next day, pericytes were incubated with a fluorescent secondary antibody (1:200, Invitrogen, US) for 2 h at room temperature. Images were taken by a fluorescence microscope (Zeiss, Germany).

### Transfection of pericytes

A lentivirus expressing the full-length sequence of the Notch1 coding region was prepared as previously described^23^. Cultured pericytes were then transfected with the lentiviral vector according to the manual instruction. The cell density was adjusted to 2 × 10^6^ cells per well in a 6-well plate. Cells were continuously cultured on conditional medium after transfection. For the knockdown of PDGFRβ or ROCK1, short hairpin RNA (shRNA) sequences were synthesized by Gimma Gene (China) based on the antisense strand of the gene coding region and were incorporated into lentiviral vectors. A scramble shRNA was used as the negative control group (sh-NC). The sequences were as follows:

sh-*ROCK1*: 5’- CCGGGCCAGCAAAGAGAGTGATATTCTCGAGAATATCACTCTCTTTGCTGGCTTTTT-3’;

sh- *PDGFRβ*: 5’- CCGGGCAACTTTGATCAACGAGTCTCTCGAGAGACTCGTTGATCAAAGTTGCTTTTTTG 3’;

sh-NC:5’- CTCCGAACGTGTCACGTTTCAAGAGAACGTGACACGTTCGGAG-3’.

### Immunohistochemistry (IHC) staining

Human lung tissues from IPF patients and normal subjects were fixed in 4% paraformaldehyde, embedded in paraffin, and sectioned into 5 μm slices. Tissue slices were first dewaxed and rehydrated using xylene followed by graded ethanol. After antigen retrieval, tissues were rinsed in deionized water. Sections were then blocked in 5% bovine serum albumin for 60 min. Tissues were then rinsed three times in PBS and were incubated with primary antibodies against Notch1, PDGFRβ, and ROCK1 (1:250, Cell Signaling Technology, USA) at 4 °C overnight. On the next day, the excess primary antibody was rinsed, and the tissues were incubated with a secondary antibody for 60 min, followed by the application of the DAB chromogenic substrate. After they were rinsed in deionized water, tissue slices were dehydrated in gradient ethanol and mounted with coverslips. Images were taken under a bright field microscope.

### MTT assay for cell proliferation

The MTT assay was employed to examine the effect of Notch1 overexpression on the proliferation of the pericytes. Transfected pericytes were maintained at 25,000 cells per well overnight at 37 °C. After a certain period of time (0, 2, 48, and 72 h), cells were washed with PBS, and 50 μL MTT solution (2 mg/mL, Invitrogen, US) were applied for a 3 h incubation. The medium was subsequently removed, and the cells were lysed with 200 μL DMSO. Cells were incubated at room temperature until the violet crystals were completely dissolved. The absorbance values at 490 nm wavelength were measured. Cell viability was then calculated against that of nontreated cells.

### Flow cytometry for cell cycle assay

The cell cycle of pericytes was further examined by flow cytometry assay. Cells were first suspended in 70% ethanol and incubated at 4 °C for 2 h. Cells were then washed with PBS and incubated with 1 μg/mL Propidium Iodide (PI, Invitrogen, US) for 15–30 min at 25 °C. The cell cycle was analyzed by flow cytometry based on the fluorescence intensity of PI in each phase.

### RNA extraction and RT-qPCR

Total RNA was isolated using TRIzol reagent according to the manual’s instruction. The cDNA was synthesized using a random primer and reverse transcription kit (Takara, Japan). Quantitative PCR was performed using a SYBR Green kit (Exiqon, Denmark) under the following conditions: denature for 30 s at 94 °C, followed by 30 cycles each consisting of 94 °C denaturation for 30 s, 60 °C annealing for 30 s and 72 °C extension for 30 s. The relative expression level of the transcripts was analyzed by the 2^-△△Ct^ approach. Primers were designed based on the coding regions of the targeted genes. The primer sequence is shown in Table 1.

**Table 1:** The sequences for RT-qPCR prime

Gene name	Species	Sequence	
		Sense (5’–3’)	Antisense (5’–3’)
*Notch1*	human	ATCTCTTGGCTCAGGCATCG	AGCGCAATGTGCGTTCAAAA
*Desmin*	human	GACCGGAAGCATGTGCTATATCT	GTTTGCCATGTCGCCCATTA
NG2	human	TGCGGAGTTCCGACATGATT	CAGAGGTCACTTGACAGGGC
α-SMA	human	TATCCCCGGGACTAAGACGGG	CAGAGCCCAGAGCCATTGTC
collagen I	human	ATGTACCAGCCACTTCGTCC	TGGCTCCTGTAGATACGCCT
PDGFRβ	human	CAGCTCTGGCCCTCAAAGG	CCAGAAAAGCCACGTTGGTG
ROCK1	human	CACCTCTCGCTTCCAGCTTA	ACTATCGGAGGGGAAATCGC
GAPDH	human	AGCCCAAGATGCCCTTCAGT	AGCCCAAGATGCCCTTCAGT
*Notch1*	mouse	TCAGTGGCCCTAATTGCCAG	CATGGCGACATGGGCTTTTC
*Desmin*	mouse	ATGAGGGAGCTGGAGGATCG	GGCTGGTTTCTCGGAAGTTG
NG2	mouse	ACAGACGCCTTTGTTCTGCT	GTCTTCTGGGCCCGAATCAT
α-SMA	mouse	CGAGCGTGAGATTGTCCGT	CCCCTGACAGGACGTTGTT
collagen I	mouse	CCCAGCCGCAAAGAGTCTAC	AGCATACCTCGGGTTTCCAC
PDGFRβ	mouse	CCTTCTCCAGTGTGCTGACA	TCATGTAGCGTCACCTCCAG
ROCK1	mouse	CTGGGAAGAAAGGGACATCA	TTCAGGCACGTCATAGTTGC

### Western blotting

We used Western blotting to measure the protein expression levels of PDGFRβ, ROCK1, NG2, α-SMA, and collagen I. In brief, cells were harvested and digested in a lysis buffer. Total protein concentration was determined by the BCA approach using a test kit (Beyotime, China). Proteins were resolved on 10% SDS-PAGE for separation and were transferred to a PVDF membrane. The membrane was blocked in 5% milk powder with 0.1% Tween-20 and was incubated overnight with primary antibodies, including anti-PDGFR-β (#3169, 1:2500, Cell Signaling Technology, US), NG2 (SC-20162, 1:2000, Santa Cruz, CA), CD13 (ab108382, 1:3000, Abcam, US), and α-SMA (ab5694, 1:3000, Abcam, US) at 4 °C. After washing, the membrane was incubated with HRP-conjugated secondary antibody for 2 h. Protein bands were visualized by enhanced chemiluminescence reagents, and images were taken and analyzed by an imaging system.

### Generation of pulmonary fibrosis mouse model

We previously have successfully generated a mouse pulmonary fibrosis model^24^. The animal study was preapproved by the ethical committee of the Hunan Cancer Hospital (license numbers: 201703). In brief, 10% chloral hydrate (3 mL/kg body weight) was injected into the peritoneal cavity of mice for anesthesia. A middle incision was made in the skin of the neck to expose the trachea, into which 0.2 ml bleomycin (5 mg/kg, Sigma, US) was applied. The mouse was then placed in a vertical position with rotation to evenly distribute the drug. Mice were sacrificed at 7, 14, and 21 days after injection. Half of the lung tissues were fixed in formaldehyde for paraffin embedding and further staining. The other half of the lung tissues were kept in liquid nitrogen for further use.

### Histological staining

Mouse lung tissues were fixed in 10% buffered formalin solution and were embedded in paraffin. Tissues were sectioned into 5 μm slices and were examined with hematoxylin-eosin (HE) staining according to standard protocols^25^. HE staining was used to examine the general morphology of tissues by their differentiated nuclei and cytoplasm staining. In brief, sections were stained in hematoxylin working solution for 10 min and were then stained in aniline blue solution for 5 min, followed by 1% acetic acid solution for 2 min. Masson’s trichrome staining was performed to examine the morphology of connective tissues due to its dark staining of collagen fibers. Similar studies for collagen expression were performed under Sirius Red staining, in which tissues slices were immersed in a 1% Sirius red/saturated picric acid solution for 1 h, followed by two rinses of 0.5% acetic acid^26^.

### Statistical analysis

Each experiment was performed three times. All data were presented as their means ± standard deviation (SD). All statistical analysis was performed using SPSS18.0 software. Comparisons of two groups were performed using the two-tailed unpaired Student’s *t*-test. A one-way ANOVA was used for comparisons among multiple groups, and multiple comparisons were further performed using a post hoc Tukey’s test. Statistical significance was defined when *p* < 0.05 (*p < 0.05, **p < 0.01, and ***p < 0.001).

## Results

### Upregulation of Notch1 and PDGFRβ in pulmonary fibrosis tissues

**Fig. 1:**
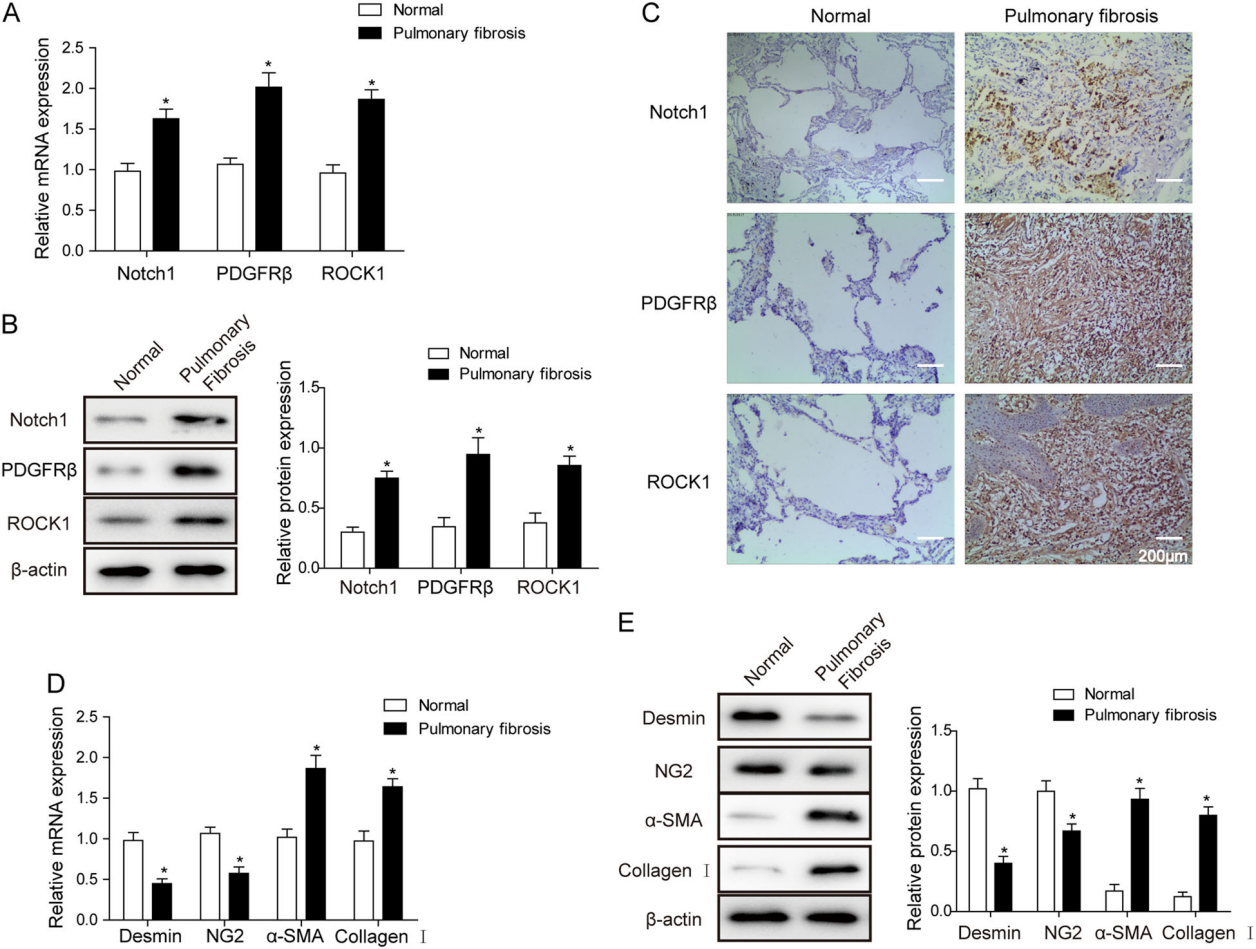
Notch1 upregulation in pulmonary fibrosis tissues. **a** Relative mRNA expression level of the Notch1 signaling pathway in normal and fibrotic tissues. **b** Western blot for Notch1, PDGFRβ, and ROCK1 proteins in normal and fibrotic tissues. β-actin was used as an internal control. **c** IHC staining for Notch1, PDGFR, and ROCK1 in lung tissues. **d** Relative mRNA level for the desmin, NG2, α-SMA, and collagen I genes in tissues. **e** Western blot for cell surface marker proteins. The means ± SD in the graph present the relative levels from three replicates. *p < 0.05

We first examined the expression of Notch1 and PDGFRβ in the lung tissues of IPF patients. First, the quantification using RT-qPCR and western blotting revealed an upregulation of transcripts (Fig. 1a) and proteins (Fig. 1b) in the fibrotic pulmonary tissues. We also found with IHC staining that both Notch1 and PDGFRβ expression levels were significantly elevated compared to those of the normal control tissues (Fig. 1c). We further examined the expression of desmin and NG2, which are marker proteins of pericytes, along with the myofibroblast cell markers α-SMA and collagen I. The results showed downregulation of desmin and NG2 plus upregulation of α-SMA and collagen I in fibrotic tissues (Figs. 1d, e), strongly indicating the acceleration of PMT in lung fibrosis.

### Notch1 overexpression facilitates pericyte proliferation and differentiation

**Fig. 2:**
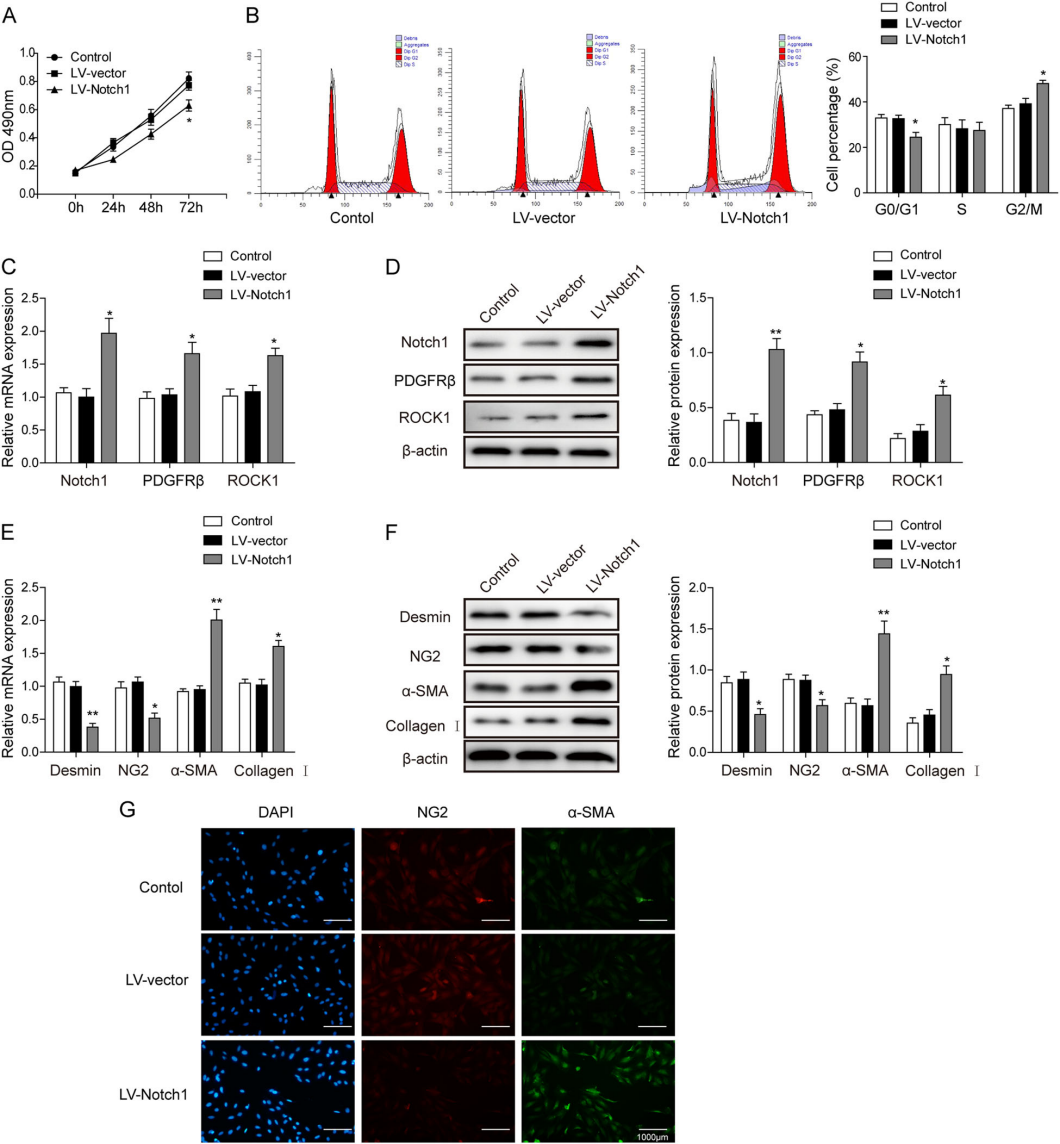
Notch1 mediates pericyte proliferation and differentiation. **a** MTT assay of the pericyte cell viability after transfection with the lv-vector and lv-Notch1, compared with that of the control cells. **b** Flow cytometry for the cell cycle analysis of pericyte cells. **c** Relative mRNA expressions of the Notch1, PDGFRβ, and ROCK1 proteins in control, lv-vector, and lv-Notch1 cells. **d** Western blot analysis showing the protein levels of Notch1, PDGFRβ, and ROCK1 in control, lv-vector, and lv-Notch1 pericyte cells. **e** Relative mRNA levels for desmin, NG2, α-SMA, and collagen I genes in control, lv-vector, and lv-Notch1 pericyte cells. **f** Western blot analysis showing the protein levels of desmin, NG2, α-SMA, and collagen I in control, lv-vector and lv-Notch1 pericyte cells. **g** Immunofluorescent staining for cell surface markers indicating pericyte differentiation. The means ± SD in the graph present the relative levels from three replicates. *p < 0.05

To further investigate the effect of Notch1 on the proliferation and differentiation of pericytes, we first examined cell behavior after overexpressing Notch1 in vitro. We found with immunofluorescent staining that more than 90% of separated pericytes expressed the specific cell markers PDGFRβ and CD13 (Supplementary Fig 1), suggesting a satisfactory purity of the cell separation. Using the MTT assay, we found that the overexpression of Notch1 effectively facilitated the proliferation of pericytes (Fig. 2a). The flow cytometry assay showed an elevated G2/M phase ratio in pericytes with Notch1 overexpression (Fig. 2b). Consistent with Fig. 1a–c, RT-qPCR and western blotting found an upregulation of PDGFRβ and ROCK1 with Notch1 overexpression (Figs 2c, d). We further examined the PMT phenomenon under Notch1 overexpression. Using similar approaches, we found the suppressed expression of desmin or NG2 and a potentiated expression of α-SMA and collagen I (Figs 2e, f). These results received further support from the results of the immunofluorescent staining, which found NG2 downregulation and α-SMA upregulation in Notch1-overexpressed pericytes (Fig. 2g). These results collectively suggest that Notch1 facilitates the proliferation of pericytes and their differentiation toward myofibroblasts.

### PDGFRβ is downstream of Notch1-mediated pericyte proliferation and differentiation

**Fig. 3:**
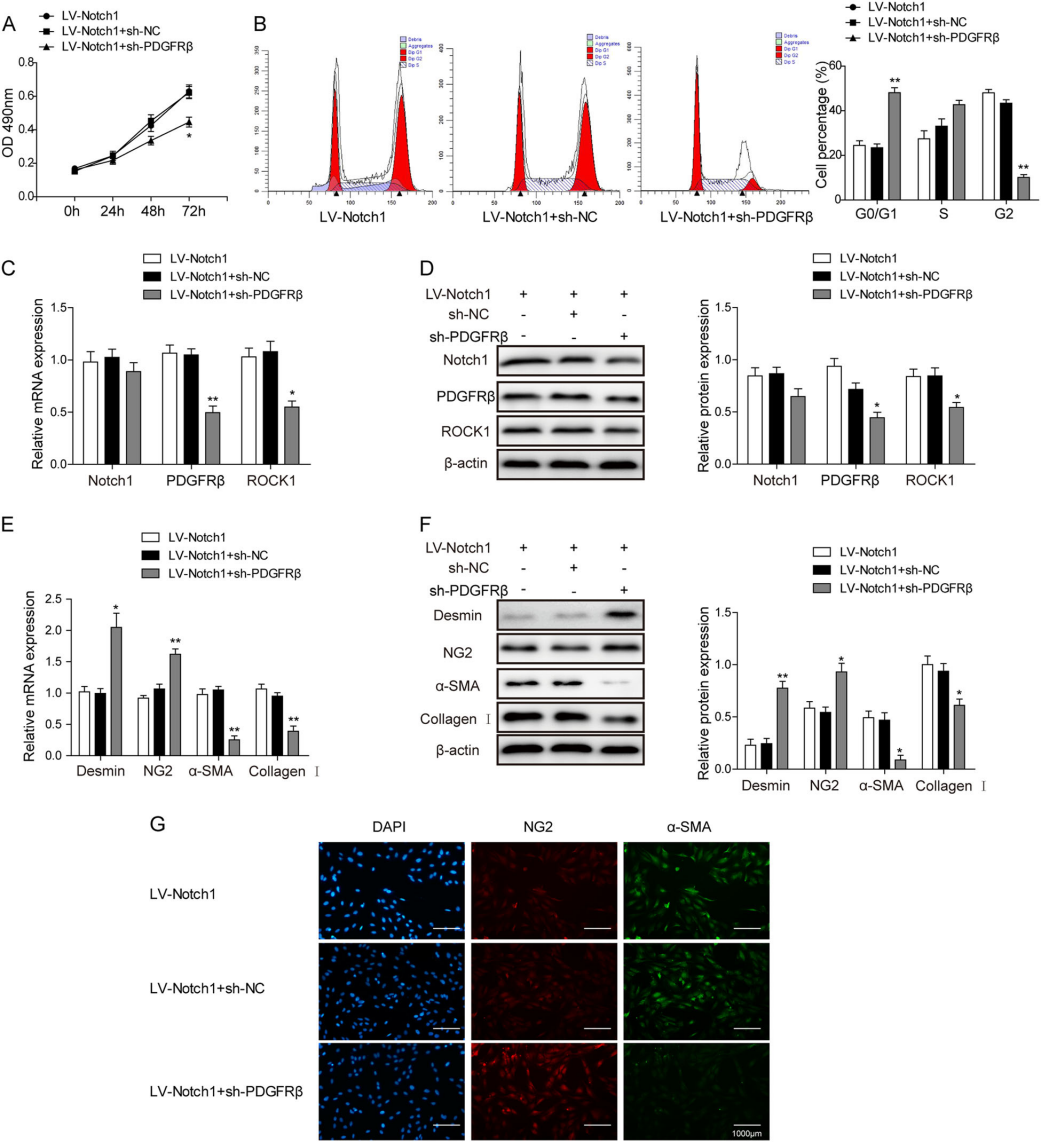
PDGFR is involved in Notch1-mediated pericyte behaviors. **a** The MTT assay for cell viability after PDGFRβ knockdown in LV-Notch1 cells (LV-Notch1 + sh-PDGFRβ) compared with that of the LV-Notch1 + sh-NC and LV-Notch1 groups. **b** Flow cytometry for cell cycle analysis of pericytes from LV-Notch1, LV-Notch1 + sh-NC, and LV-Notch1 + sh-PDGFRβ groups. **c** Relative mRNA expression of Notch1, PDGFRβ, and ROCK1 in the LV-Notch1, LV-Notch1 + sh-NC, and LV-Notch1 + sh-PDGFRβ groups. **d** Western blot for Notch1, PDGFRβ, and ROCK1 protein expression. **e** Relative mRNA levels for the pericyte differentiation marker genes. **g** Western blot analysis showing the protein levels of desmin, NG2, α-SMA, and collagen I in the LV-Notch1, LV-Notch1 + sh-NC, and LV-Notch1 + sh-PDGFRβ groups. **f** Immunofluorescent staining for cell surface markers indicating pericyte differentiation. The means ± SD in the graph present the relative levels from three replicates. *p < 0.05

To investigate the possible signaling pathway that is involved in the Notch1-initiated behavior of pericytes, we used shRNA knockdown of PDGFRβ to investigate its role in pericyte proliferation and differentiation. The MTT assay first revealed a weakened Notch1-induced pericyte proliferation after PDGFRβ downregulation (Fig. 3a). Consistent with these results, flow cytometry showed higher levels of cell arrest at the G0/G1 phase after PDGFRβ knockdown (Fig. 3b). Both the RT-qPCR and western blotting analyses showed downregulation of ROCK1 after PDGFRβ knockdown, while Notch1 expression remained unchanged after the same treatment (Figs. 3c, d). These results convincingly showed that PDGFRβ is located downstream of Notch1 to mediate pericyte proliferation. We further examined the expression of pericyte and myofibroblast cell markers. The results showed that PDGFRβ knockdown efficiently increased the expression of the pericyte markers desmin and NG2, while the myofibroblast markers α-SMA and collagen I were upregulated (Figs. 3e–g). These results collectively showed that PDGFRβ played crucial roles in mediating the Notch1-induced PMT phenomenon.

### ROCK1 mediates PMT behavior

**Fig. 4:**
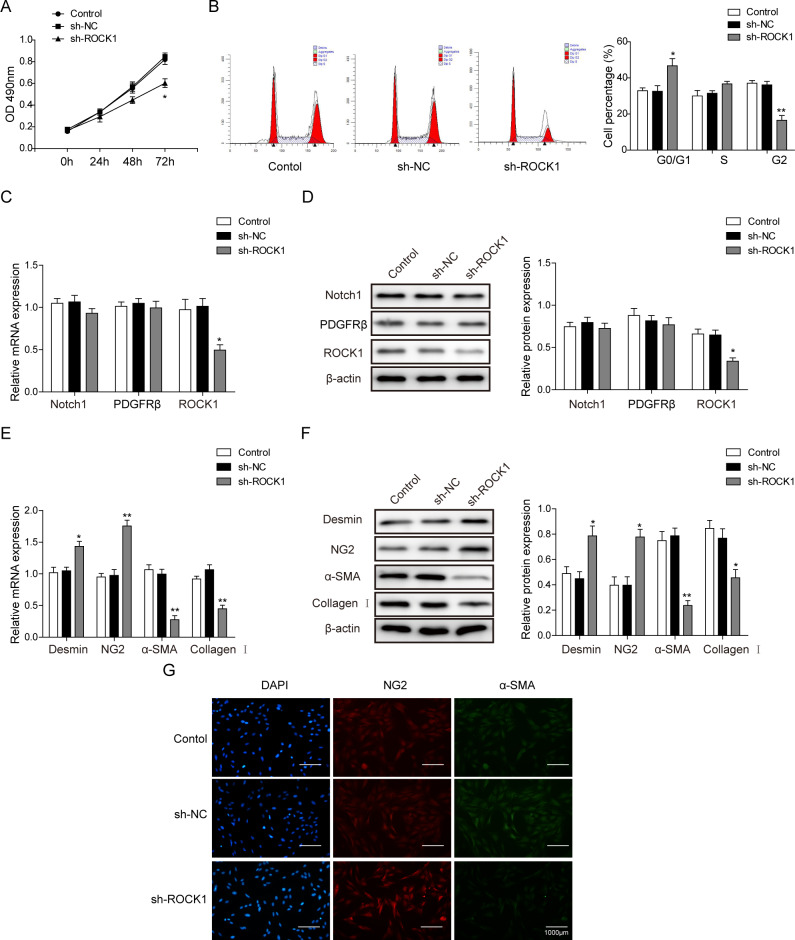
ROCK1 is downstream of Notch1/PDGFR to mediate pericyte proliferation. **a** The MTT assay for cell viability after ROCK1 knockdown. **b** Flow cytometry for cell cycle analysis of pericytes from the control, sh-NC, and sh-ROCK1 pericyte cells. **c** Relative mRNA expression of Notch1, PDGFRβ, and ROCK1. **d** Western blot for Notch1, PDGFRβ, and ROCK1 protein expression. **e** Relative mRNA expression for cell marker genes. **f** Western blot for quantification of cell markers. **g** Immunofluorescent staining for cell surface markers indicating pericyte differentiation. The means ± SD in the graph present the relative levels from three replicates. *p < 0.05

In addition, we examined the role of ROCK1 in the process of pericyte proliferation and differentiation. With the shRNA knockdown of ROCK1, we used the MTT assay to find that ROCK1 was involved in pericyte proliferation (Fig. 4a). Flow cytometry also showed that ROCK1 knockdown elevated the ratio of pericytes at the G0/G1 phase (Fig. 4b). No significant changes in Notch1 or PDGFRβ were found after ROCK1 knockdown (Figs. 4c, d), illustrating that ROCK1 was downstream of the Notch1-PDGFRβ axis. Consistent with these results, ROCK1 knockdown elevated the pericyte markers desmin and NG2 and decreased the levels of the myofibroblast markers α-SMA and collagen I (Figs. 4e–g). These results showed that Notch1-mediated pericyte proliferation and PMT via the PDGFRβ-ROCK1 pathway.

### Notch1 inhibitor alleviates pulmonary fibrosis in vivo

**Fig. 5:**
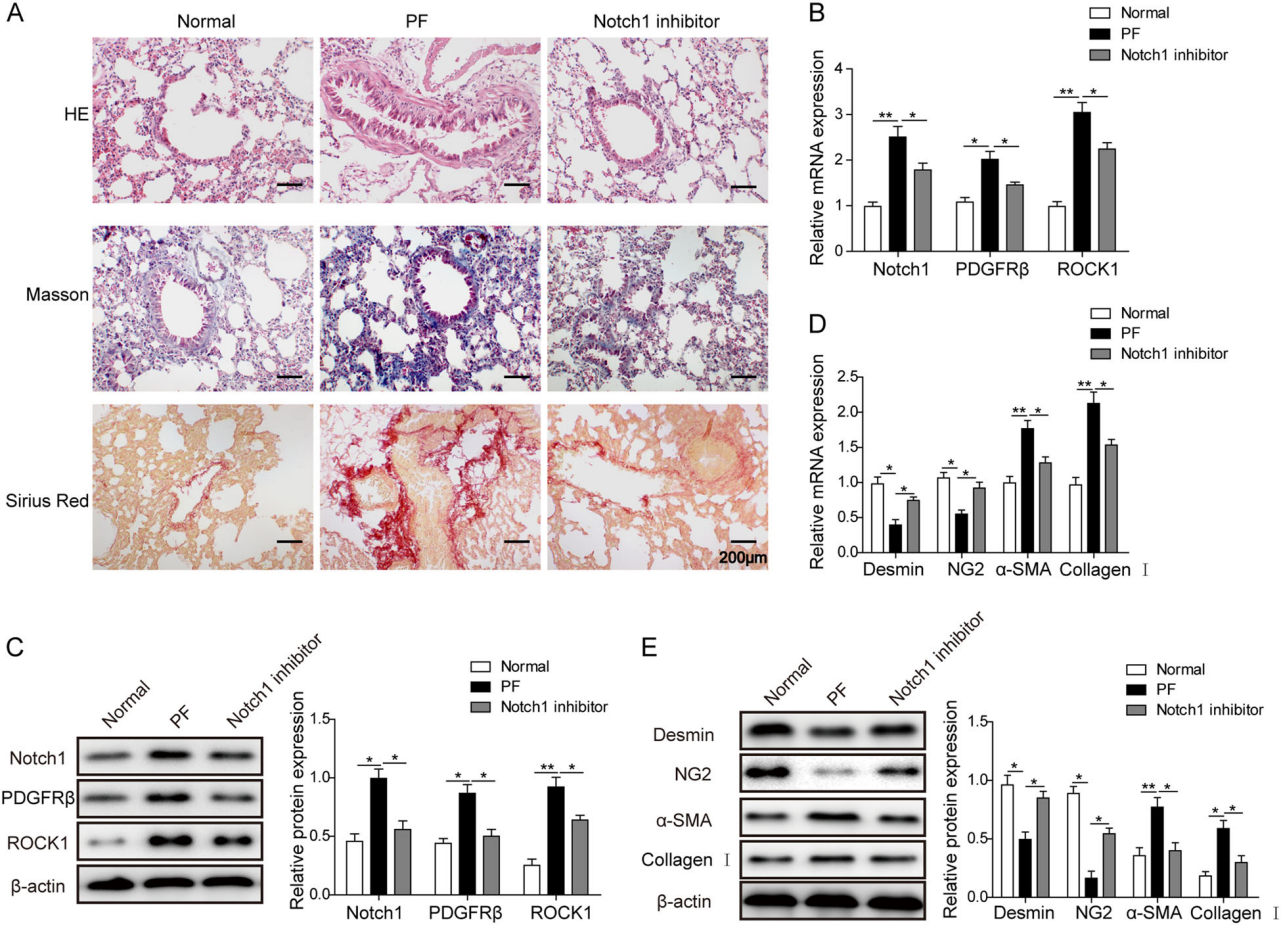
Notch1 inhibitor alleviated mouse pulmonary fibrosis in vivo. **a** Tissue staining of mouse lungs by the HE, Masson and Sirius Red methods. **b** Relative mRNA expression of the Notch1, PDGFRβ, and ROCK1 genes in the normal, PF and Notch1 inhibitor groups. **c** Western blot for the expression of proteins in the Notch1 signaling pathway. **d** Relative mRNA for desmin, NG2, α-SMA, and collagen I genes in the three groups. **e** Western blot analysis showing the protein levels of desmin, NG2, α-SMA, and collagen I in the normal, PF and Notch1 inhibitor tissues. The means ± SD in the graph present the relative levels from three replicates. *p < 0.05

To substantiate the effect of the Notch1/PDGFRβ/ROCK1 axis on pericyte differentiation and pulmonary fibrosis, we generated a mouse pulmonary fibrosis model in which the Notch1 signaling pathway inhibitor DAPT was used as a treatment. Using HE staining, we found that tissues in the pulmonary fibrosis (PF) model presented with prominent tissue fibrosis, and this could be partially alleviated by the Notch1 inhibitor (Fig. 5a). Similar results were obtained from the Masson staining, which clearly showed an intense expression of collagen fiber in the PF mice and an improvement after treatment with the Notch1 inhibitor. Moreover, the Sirius Red staining also showed an upregulation of collagen under the fibrotic condition but a partial rescuing effects after Notch1 inhibition (Fig. 5a). Both RT-qPCR and western blotting found that the inhibition of Notch signaling pathway downregulated PDGFRβ and ROCK1 expression (Figs. 5b, c). In addition, we measured both pericyte and myofibroblast cell markers. The results showed that treatment with the Notch1 inhibitor increased the expression of the pericyte markers desmin or NG2 and decreased the expression of the myofibroblast surface markers α-SMA and collagen I (Figs. 5d, e). These results provide further in vivo evidence to show that the Notch1/PDGFRβ/ROCK1 signaling pathway is involved in pulmonary fibrosis pathogenesis, and the inhibition of the Notch1 signaling pathway could suppress the PMT phenotype to alleviate the condition of pulmonary fibrosis.

## Discussion

In this study, we first investigated the expression level of Notch1 signaling pathway molecules in lung tissues samples that were obtained from IPF patients. Both immunostaining and gene expression assays showed an upregulation of Notch1, PDGFRβ, and ROCK1 in the patient group. These results have been previously reported in rodent models. For example, in bleomycin-induced rat pulmonary fibrosis, the level of Notch1 is upregulated^12^. As the downstream effector of Notch1, the level of PDGFRβ has been shown to be potentiated in a mouse lung fibrosis model^27^. Increased ROCK1 expression is also widely observed in pulmonary fibrosis conditions. ROCK1 is also believed to contribute to pulmonary fibrosis in animal models^28^. More importantly, the level of ROCK1 has been demonstrated to function as a clinical progression marker for IPF^21^. All of these studies have shown the involvement of Notch1 and its downstream signaling pathway in the development of pulmonary fibrosis, suggesting the potency of using these molecules as the targets for potential drug intervention.

In studying IPF, it is critical to investigate the role of the lung pericyte, whose abnormal proliferation and maturation has been implicated in the pathogenesis of the disease. We thus studied the effect of Notch1 on pericyte behaviors, including its proliferation and differentiation, toward myofibroblasts. We found that Notch1 positively modulated cell proliferation and differentiation. Similar results have been previously found in kidney tissues, in which Notch1 is believed to be necessary for the development of the pericyte population^29^. Moreover, Notch1 is believed to mediate the PMT process in vascular tissues^9^. However, this study is the first time that lung pericyte proliferation was found to be mediated by Notch1.

Furthermore, we knocked down PDGFRβ and ROCK1 expression in lung pericytes and studied the effect of PDGFRβ and ROCK1 on the pericyte transition. Our results showed that Notch1 modulated the pericyte transition via PDGFRβ/ROCK1 signaling pathway. As the downstream effector of Notch1, the role of PDGFRβ has been recognized in the process of renal pericyte proliferation and fibrosis development^30^. Similar results can be found in the mouse brain, where PDGFRβ is found to mediate the transition of vascular pericytes into glioma cells^31^. These results collectively suggest the functional role of Notch1-PDGFRβ in pericyte proliferation and differentiation to further the pathogenesis of fibrosis. Downstream signaling molecules of PDGFRβ include Ras, PI3K, and PLC. Through these pathways, ROCK1 is known to exert certain roles in pulmonary fibrosis^21, 28, 32^. However, no study has been performed on the role of ROCK1 in pericyte proliferation or differentiation. We thus expect that ROCK1 works in a synergistic manner with Notch1 to mediate pericyte behavior, and such a proposed model has received support from our experimental results.

After illustrating the molecular pathway of Notch1 in mediating pericyte proliferation and cell behavior in vitro, we further tested the effect of Notch1 on the pathogenesis of pulmonary fibrosis with a mouse model. Our results showed that the Notch1 inhibitor effectively alleviated pulmonary fibrosis, as supported by an improved histological phenotype and decreased PMT activity. In cultured A549 cells, the epithelial-mesenchymal transition (EMT) progress was remarkably retarded by the Notch1 inhibitor, indicating its potency in treating pulmonary fibrosis^33^. Although no direct in vivo study has been performed using a Notch1 inhibitor to treat pulmonary fibrosis in either animal models or in humans, the Notch1 signaling pathway has been implicated the fibrotic process in tissues such as the liver^34^ and in systemic sclerosis^35^. Based on this information and the obtained promising results for the Notch1 inhibitor in mouse pulmonary fibrosis, we thus proposed that the Notch1 inhibitor may work as a potent drug candidate in treating IPF. Although similar effects can be obtained using a PDGFRβ or ROCK1 inhibitor to attenuate pulmonary fibrosis^36^, the application of a Notch1 inhibitor further demonstrated that Notch1 is upstream of the PDGFRβ-ROCK1 axis to mediate pericyte behaviors.

In summary, our study described the overactivation of the Notch1/PDGFRβ/ROCK1 pathway in IPF patient lung tissues, illustrating the facilitating role of the Notch1 pathway in pericyte proliferation and their differentiation toward myofibroblasts, which may further aggravate tissue fibrosis. Moreover, we reported for the first time the effectiveness of the Notch1 inhibitor in alleviating mouse lung fibrosis via suppressing pericyte proliferation and transition. Our results provide more insights for the pathogenesis mechanism that underlies IPF and provide a promising treatment approach for lung fibrosis.

## Supplementary information

Supplementary Materials
