# Author Correction: Notch1 promotes the pericyte-myofibroblast transition in idiopathic pulmonary fibrosis through the PDGFR/ROCK1 signal pathway

**DOI:** 10.1038/s12276-025-01477-2

**Published:** 2025-06-24

**Authors:** Yi-Chun Wang, Qiong Chen, Jun-Ming Luo, Jing Nie, Qing-He Meng, Wei Shuai, Han Xie, Jia-Mei Xia, Hui Wang

**Affiliations:** 1 Department of Critical Care Medicine, The Third Affiliated Hospital of Guangzhou Medical University, No. 63, Duobao Road, Guangzhou, 510150 P. R. China; 2 Department of Critical Care Medicine, Hunan Cancer Hospital, Changsha, 410013 P. R. China; 3 Department of Surgery, SUNY Upstate Medical University, Syracuse, NY 13210 USA; 4 Department of Thoracic Radiotherapy, Hunan Cancer Hospital, Changsha, 410013 P. R. China

Correction to: *Experimental and Molecular Medicine* 10.1038/s12276-019-0228-0, published online 20 March 2019

In this article, the second group picture (sh-NC) in Fig. 4G appeared as incorrectly placed and should have appeared as the revised picture below.

**Original Figure 4G.** 
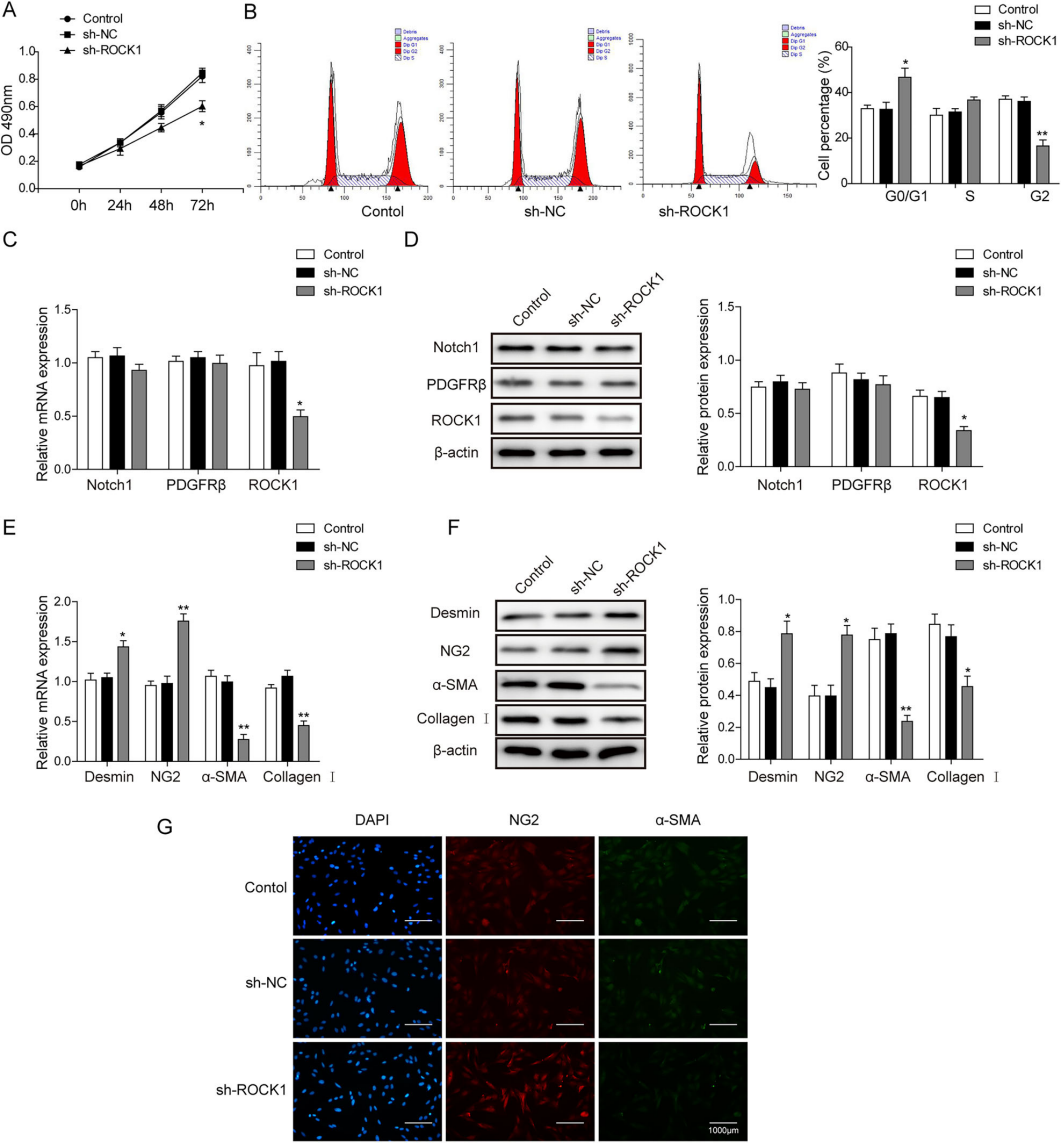


**Corrected Fig. 4G.** 
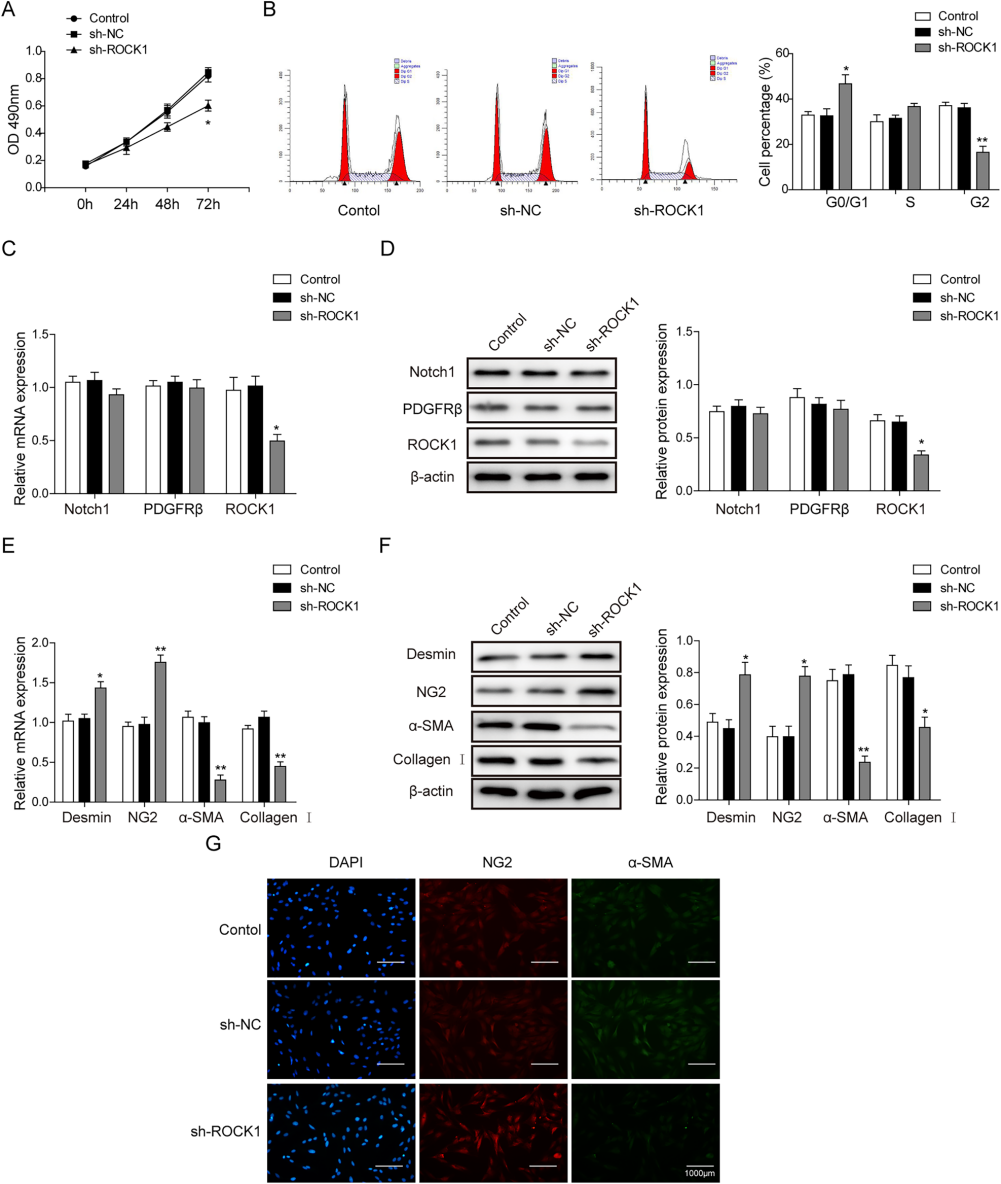


The original article has been corrected.

