# Supplementary material for: Notch1 promotes the pericyte-myofibroblast transition in idiopathic pulmonary fibrosis through the PDGFR/ROCK1 signal pathway
Source: Exp Mol Med. 2019 Mar 20;51(3):1–11. doi: 10.1038/s12276-019-0228-0 (PMC6430797; doi:10.1038/s12276-019-0228-0)

**Supplementary material**

**Figure 1. Description of purified pericytes.** Immunofluorescent staining for PDGFRβ (Red) CD13 (Green) and DAPI (Blue) in separated pericytes.


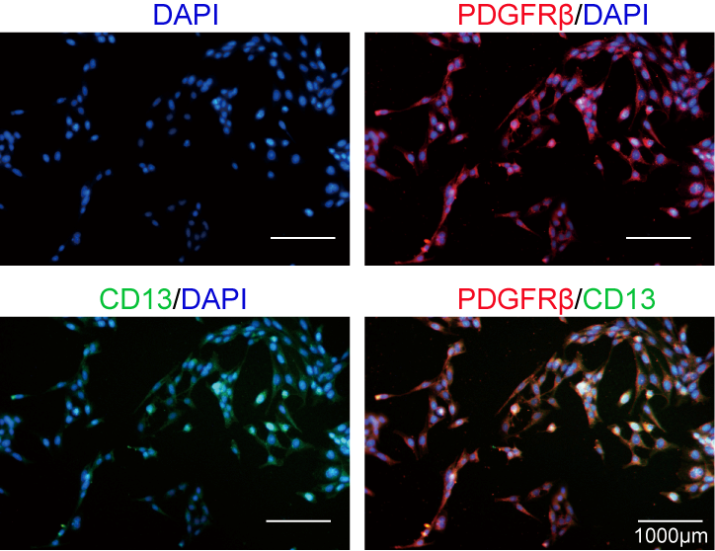

Supplement: Supplementary file 1 — Supplementary Materials [file 12276_2019_228_MOESM1_ESM.docx]
